# MicroRNA-338-3p as a novel therapeutic target for intervertebral disc degeneration

**DOI:** 10.1038/s12276-021-00662-3

**Published:** 2021-09-16

**Authors:** Hua Jiang, Abu Moro, Jiaqi Wang, Dihua Meng, Xinli Zhan, Qingjun Wei

**Affiliations:** 1 Division of Spine Surgery, The First Affiliated Hospital of Guangxi Medical University, Nanning, China; 2 Department of Orthopaedic Surgery, The First Affiliated Hospital of Guangxi Medical University, Nanning, China

**Keywords:** Genetic markers, Translational research, Gene therapy

## Abstract

Recent studies have demonstrated the pivotal role played by microRNAs (miRNAs) in the etiopathogenesis of intervertebral disc degeneration (IDD). The study of miRNA intervention in IDD models may promote the advancement of miRNA-based therapeutic strategies. The aim of the current study was to investigate whether intradiscal delivery of miRNA can attenuate IDD development. Our results showed that miR-338-3p expression was significantly increased in the nucleus pulposus (NP) of patients with IDD. Moreover, there was a statistically significant positive correlation between the expression level of miR-338-3p and the severity of IDD. Our functional studies showed that miR-338-3p significantly influenced the expression of extracellular matrix synthesis genes, as well as the proliferation and apoptosis of NP cells. Mechanistically, miR-338-3p aggravated IDD progression by directly targeting SIRT6, a negative regulator of the MAPK/ERK pathway. Intradiscal injection of antagomir-338-3p significantly decelerated IDD development in mouse models. Our study is the first to identify miR-338-3p as a mediator of IDD and thus may be a promising target for rescuing IDD.

## Spine disease: how to halt disc degradation

Development of intervertebral disc degeneration (IDD) is driven by a microRNA, a short noncoding segment of genetic material, called miR-338-3p, and blocking it may offer a path to better treatments. In IDD, a common spine disorder, the cushioning discs between vertebrae degenerate, leading to pain and loss of mobility. Current treatments address symptoms, not the root causes. Micro-RNAs have emerged as key contributors to skeletal disorders, and Qingjun Wei and Hua Jiang at Guangxi Medical University, Nanning, China, investigated their role in IDD. Screening of microRNAs in IDD patients showed that miR-338-3p levels were significantly elevated and correlated with IDD severity. Inhibiting miR-338-3p in cultured disc cells boosted their proliferation, and administering the inhibitor to a mouse model suppressed IDD development. These results may help in identifying new treatments for this common and debilitating condition.

## Introduction

Intervertebral disc degeneration (IDD, OMIM 603932), one of most common spine disorders, is characterized by the progressive loss of disc space, endplate sclerosis, and low back pain (LBP)^1^. It is among the main factors affecting quality of life and exacts a huge burden on the global healthcare system^2,3^. An intervertebral disc is composed of the central nucleus pulposus (NP) and the outer annulus fibrosus (AF). The NP is involved in maintaining the homeostasis of proteoglycan and type-II collagen (Col II) in the extracellular matrix (ECM)^4^. A hallmark of IDD is the degradation of proteoglycans in the NP, leading to decreased disc height and reduced biomechanical function^5,6^. Clinically, IDD causes disc herniation and sciatica, which are associated with back pain and chronic disability. With regard to therapeutics, IDD presents with a complex of degenerative diseases. Conservative and surgical treatments have limited long-term effects in many IDD cases, as these methods only target the clinical symptoms but do not focus on the etiology of IDD^7^. Recent advancements in gene therapy have opened an avenue for the possible prevention of IDD development at the transcriptional or post-transcriptional levels^8–11^. There is therefore the need for a more in-depth understanding of IDD to facilitate the development of new therapeutic strategies^12^.

MicroRNAs (miRNAs), a group of endogenous noncoding RNAs, have emerged as key regulators in the development of skeletal pathophysiology^13^. Studies have reported that they bind to the 3′ untranslated region (3′ UTR) of target mRNAs, resulting in the modulation of gene expression^14^. Accumulating evidence has demonstrated that miRNAs act as central modulators in essential cellular processes, including cellular proliferation and apoptosis^15,16^. Notably, dysregulation of miRNAs has been reported to be related to various degenerative diseases in humans^17–20^. Differential expression patterns of miRNAs have been noted in the pathogenesis of IDD. Several miRNAs, such as miR-155, miR-93, and miR-141, were significantly downregulated or upregulated in patients with IDD and thus were identified as targets of IDD-associated gene expression ranging from matrix-degrading enzymes to proinflammatory cytokines^21–24^. In the current study, we performed a comprehensive screening of 2000 miRNAs in human NP tissue. miRNA expression profiling led to the identification of miR-338-3p as substantially increased in degenerative NP tissue compared with control NP tissue. Moreover, miR-338-3p expression was positively correlated with the Pfirrmann grade of IDD. Our functional studies revealed that miR-338-3p induced IDD by promoting the catabolism of NP cells and NP cell apoptosis via the sirtuin 6 (SIRT6)/mitogen-activated protein kinase (MAPK)/extracellular signal-regulated protein kinase (ERK) pathway. Our in vivo study investigating the possible effects of intradiscal injection of an miR-338-3p inhibitor in mouse models revealed that this microRNA exerted an NP-protective effect. Thus, our findings provide, for the first time, crucial evidence that miR-338-3p holds promise as a possible target for the prevention of IDD.

## Materials and methods

### Patient samples

Nucleus pulpous (NP) tissues were collected from 110 patients with IDD who underwent lumbar microdiscectomy (mean age 56.3 ± 9.1 years). All IDD patients were clinically diagnosed by two qualified spine surgeons via physical examination and MRI. On the basis of the Pfirrmann classification^25^, all patients were classified as having mild/moderate degeneration (grades 1 or 2) or serious degeneration (grades 3 or 4). Normal NP tissues were collected from 103 patients with lumbar vertebral fracture who underwent anterior spine surgery (mean age 53.7 ± 4.6 years). These trauma patients had no prior history of preoperative low back pain or IDD.

### The injury-induced IDD model and therapeutic experiment

The injury-induced IDD mouse model was established via needle puncture as previously described^23,26^. Briefly, following general anesthesia of 12-week-old C57BL/6 mice, the mouse coccygeal discs Co6/Co7 were exposed and then punctured with a 31-G syringe needle through the AF to the NP. The needle was then left in the intervertebral disc at a depth of 1.5 mm for a period of 10 s. The adjacent Co7/Co8 disc levels were not punctured, as they were used as contrast segments.

For the therapeutic tests, 20 male mice that underwent the previously described injury-induced IDD surgery were randomly divided into four groups (*n* = 5): group 1 (agomir–control); group 2 (agomir-338-3p); group 3 (antagomir–control); and group 4 (antagomir-338-3p) (the treatment group). The agomir- or antagomir-338-3p groups and their controls were acquired from RiboBio (RiboBio Co., Guangzhou, China). The mice were injected with 10 μl of solution containing agomir-338-3p, antagomir-338-3p, or their negative controls on the 1^st^, 7^th^, and 14^th^ days after needle-puncture intervention. The disc samples were then harvested at two different time periods: the 6^th^ week and 12^th^ week postintervention.

For recombinant adenovirus construction, the sequence for sirt6 from human genomic DNA, which was cloned by PCR, was inserted into the pDC311-U6-MCMV-EGFP vector (Ad-sirt6) (Hanbio Co. Ltd, Shanghai, China), and sirt6 siRNA was purchased from Life Technologies. Adenoviral vectors encoding green fluorescent protein (Ad-GFP) were used as controls. A total of 15 injury-induced IDD mice were included in the study and were randomly divided into three groups (*n* = 5). Three, seven and fourteen days after puncture intervention, the intervertebral discs were injected with Ad-sirt6 (1 × 10^9^ viral particles), Ad-GFP (1 × 10^9^ viral particles), and sirt6 siRNA, respectively. The mice were sacrificed by pentobarbital overdose eight weeks after adenovirus injection.

The ethics committee of The First Affiliated Hospital of Guangxi Medical University approved the animal experimentation protocol (2018-KY-NSFC-025), and all animal experimentations were conducted in accordance with approved guidelines.

### Cell cultures

Human NP tissues were sliced into 1-mm^3^ pieces and washed twice with 10% fetal bovine serum (PBS) (Gibco, NY, USA), followed by incubation in DMEM (Gibco, NY, USA). Subsequently, a resuspension of NP cells was cultured in DMEM, 1% penicillin–streptomycin, and 10% FBS. The cells were then cultured in an incubator at 37 °C and 5% CO_2_, dissociated from the substrate with trypsin, and split at a ratio of one to three.

### Quantitative real-time polymerase chain reaction (qRT-PCR)

TRIzol (Invitrogen Life Technologies, CA, USA) was used for total RNA extraction. The RNA templates were then synthesized into cDNA using an iScripts cDNA Synthesis kit (Quanta BioSciences, MD, USA), and GAPDH was used as the control for normalization. The isolated miRNA was then quantified using a qScript microRNA cDNA synthesis kit (Quanta BioSciences, MD, USA), with U6 snRNA used as the internal control. Then a SYBR Green real-time PCR kit (Quanta Biosciences) was used to perform qRT-PCR, with a comparative threshold cycle (ΔΔCt) adopted for the calculation of gene expression. The primer sequences are shown in Supplementary Table 1.

### miRNA microarrays and miRNA target prediction

The miRNA microarray study was conducted using an Affymetrix 4.0 miRNA Array (Thermo Fisher Scientific, MA, USA). Total RNA was obtained from six individual NP samples with or without IDD. Based on a volcano plot and fold-change filtering, we identified the differentially expressed miRNAs in the IDD samples. Candidate miRNAs were included in the analysis they met the following criteria: (1) exhibited more than a 5-fold change or less than a 0.2-fold change, and (2) differences had p-values less than 0.05. Gene Cluster software (Stanford University) and DAVID software were used for hierarchical cluster analysis and functional group analysis, respectively. TargetScanHuman (www.targetscan.org/) and microRNA.org were used to forecast the miRNA target genes and to analyze mRNA-binding sites.

### miR-338-3p and SIRT6 transfection

For overexpression or silencing of miR-338-3p, human NP cells were transfected with miR-338-3p mimics or inhibitor or their negative controls (Cat. No: 4464061 and 4464079, Life Technologies) using Lipofectamine RNAiMAX Transfection Reagent (Invitrogen). For the suppression of SIRT6 expression, transient transfection of human NP cells with SIRT6 siRNA or control siRNA (Cat. No: 116148 and 4459408, Thermo Fisher Scientific) was carried out using Lipofectamine 3000 Transfection Reagent (Invitrogen). The SIRT6 expression plasmid (pcDNA3.1(+)/SIRT6) was obtained from Invitrogen.

### Luciferase constructs and reporter assay

SIRT6 mRNA 3′ UTR fragments, including the binding sites of wild-type (wt) and mutant (mut) miR-338-3p, were inserted into a psiCHECK-2 luciferase reporter vector (Promega, WI, USA). An miR-338-3p expression plasmid or a control plasmid was then cotransfected with SIRT6 wt- or mut-3′ UTR and their respective control plasmids into human NP cells. All luciferase assays were performed using a Dual-Glo Luciferase Assay System (Promega, WI, USA), and luciferase activity was measured by a fluorescence microplate reader (BioTek, USA).

### Flow cytometry, cell counting kit-8 (CCK-8), and EdU assay

Cellular apoptosis was measured using an apoptosis kit with Annexin V-FITC/PI (Cat. No: V13242, Invitrogen), and analysis was performed with Beckman Coulter EPICS Altra (USA). The detection of cellular proliferation was performed using CCK-8 (Dojindo Laboratories, Kumamoto, Japan). Human NP cells that were transfected with different sequences (miR-338-3p mimics, control mimics, miR-338-3p inhibitor, control inhibitor, SIRT6, SIRT6 siRNA, or control siRNA) were then added into 96-well plates and cultured for 24, 48, and 72 h. Next, CCK-8 was added to the cells, which were incubated for 3 h, and the absorption was then evaluated at 450 nm. The degree of cellular proliferation was assessed by EdU assay. Human NP cells were incubated with EdU medium (Sigma-Aldrich, MO, USA), and the cells were collected and stained with Hoechst 33258.

### Fluorescence in situ hybridization (FISH)

For the analysis of human NP using FISH, digoxin-labeled miR-338-3p probes with locked nucleic acid modifications were designed and synthesized using Exiqon QIAGEN (Hilden, Germany). The fluorescence signals were detected using a FISH kit (Exiqon-QIAGEN) with a Nikon A1Si laser-scanning confocal microscope (Nikon, Tokyo, Japan) used for image analysis.

### Western blotting and coimmunoprecipitation

Proteins extracted from human NP cells were quantified using a Micro BCA protein assay kit (Cat. No: 23235, Thermo Fisher Scientific) and then equal amounts of protein samples were separated on 10% SDS/PAGE (sodium dodecyl sulfate–polyacrylamide) gels and then electroblotted onto polyvinylidene fluoride membranes (Bio-Rad Laboratories). The membranes were then incubated with the following primary antibodies: anti-Col II, anti-Aggrecan, anti-ADAMTS4, anti-MMP9, anti-Caspase-3, anti-Bax, anti-P16, anti-p-ERK1/2, anti-ERK1/2, anti-GRB2, anti-SIRT6, anti-MEK1, anti-MEK2, anti-Raf, anti-Ras, and anti-GAPDH (ab34712, ab36861, ab185722, ab38898, ab13847, ab132503, ab151303, ab214362, ab184699, ab32111, ab191385, ab178876, ab265586, ab137435, ab108602, and ab9485, respectively; Abcam, Cambridge, UK). After washing, the membranes were then treated with 1/2000 diluted secondary goat anti-rabbit antibody (Abcam, Cambridge, UK). The resulting patterns were then interpreted using Quantity One version 4.50 software (Bio-Rad, Hercules, CA, USA). Coimmunoprecipitation analysis was performed on the basis of nuclear and cytoplasmic proteins. An extraction kit (78833, Thermo Scientific) was then used to extract nuclear and cytoplasmic proteins from human NP cells, and the expression levels of GRB2 and SIRT6 were detected using anti-GRB2 (ab32111, Abcam) and anti-SIRT6 (ab191385, Abcam) antibodies.

### Immunofluorescence and TUNEL staining

Intervertebral discs were fixed, decalcified, and embedded in 10% formalin, 10% EDTA, and paraffin. Sagittal sections of disc tissue were then cut every 3 μm from the midsagittal plane. The sections were then stained with Alcian blue for the histological analysis. Masuda’s method was then used to grade the histological staining score^27^. The cultured NP cells were then fixed, permeabilized, and blocked with 4% paraformaldehyde, 0.3% Triton X-100, and 1% BSA. For the immunofluorescence analysis, the following primary antibodies were incubated with sections of cells: anti-MMP9 and anti-Col II antibodies (ab73734 and ab34712, respectively; Abcam, Cambridge, UK) at 4 °C overnight. The sections and cells were then treated with a secondary antibody for 20 min at room temperature, followed by the capture of immunofluorescence images using a Zeiss LSM780 confocal microscope (Carl Zeiss, Oberkochen, Germany). For the detection of apoptotic activity, a TUNEL assay was performed using a DeadEnd Colorimetric TUNEL System in accordance with the manufacturer’s protocol (Cat. No: G7360, Promega).

### Statistical analysis

All statistical analyses were performed using SPSS version 17.0 software (SPSS Inc., Chicago, IL). Differences between two or more groups were analyzed by two-tailed unpaired Student’s test or one-way ANOVA with Tukey’s post hoc test. A *p*-value less than 0.05 was considered statistically significant.

## Results

### Identification of differentially expressed miRNAs between IDD patients and normal controls

**Fig. 1:**
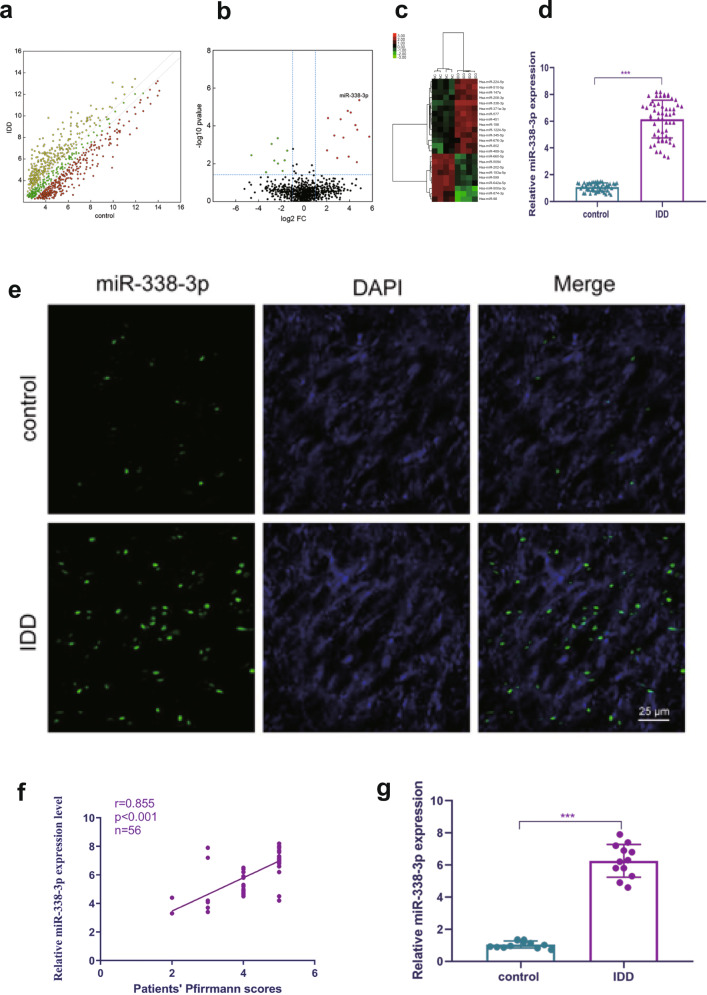
Differential expression of miRNAs between IDD patients and normal controls. **a**, **b** Scatterplot and volcano plot showing the expression profiles of miRNAs between IDD patients and normal controls. **c** Heat map illustrating 23 significantly dysregulated miRNAs in IDD patients (14 miRNAs upregulated and nine miRNAs downregulated). **d** qRT-PCR showing the high degree of expression of miR-338-3p in IDD patients compared with that of the control group. **e** FISH analysis demonstrating the upregulation of miR-338-3p in NP tissues from patients with IDD. (scale bar = 25 μm). **f** The significantly positive correlation between miR-338-3p expression and Pfirrmann grade of IDD (*n* = 56, *r* = 0.855, *P* < 0.0001). **g** The upregulation of miR-338-3p in cultured NP cells of IDD patients but not those of normal controls. ***P < 0.01.

To investigate dysregulated miRNAs, we scanned the expression profile of miRNAs based on microarrays of human NP tissues (3 IDD patients vs. 3 normal controls). The microarray revealed approximately 600 differentially expressed miRNAs between the IDD and control patients (Fig. 1a). The results of the unsupervised clustering analysis of miRNAs are shown in a volcano plot (Fig. 1b) and heat map (Fig. 1c). We identified a total of 14 upregulated and nine downregulated miRNAs that exhibited a mean fold change of more than 5-fold or less than 0.2-fold as well as a *p*-value less than 0.05. Specifically, miR-338-3p, miR-198, and miR-874-3p were found to be significantly dysregulated in the NP of IDD samples but not in that of the control samples (Supplementary Table 2). We performed qRT-PCR to identify the three most highly expressed miRNAs based on a cohort of 110 patients with IDD and 103 normal controls. Among the most highly expressed miRNAs, miR-338-3p was observed to be the most significantly upregulated in the NP tissues obtained from IDD patients (Fig. 1d). Therefore, it was selected as the candidate miRNA for further study. FISH confirmed that miR-338-3p was significantly increased in the NP of IDD patients compared to that of the control (Fig. 1e). Our results also revealed that the expression levels of miR-338-3p were positively correlated with the Pfirrmann grade of IDD (*n* = 56, *r* = 0.855, *P* < 0.01, Fig. 1f). In human NP cells, the expression levels of miR-338-3p were also found to be upregulated in IDD patients but not in normal controls (Fig. 1g).

### The effect of miR-338-3p on the proliferation and apoptosis of human NP cells

**Fig. 2:**
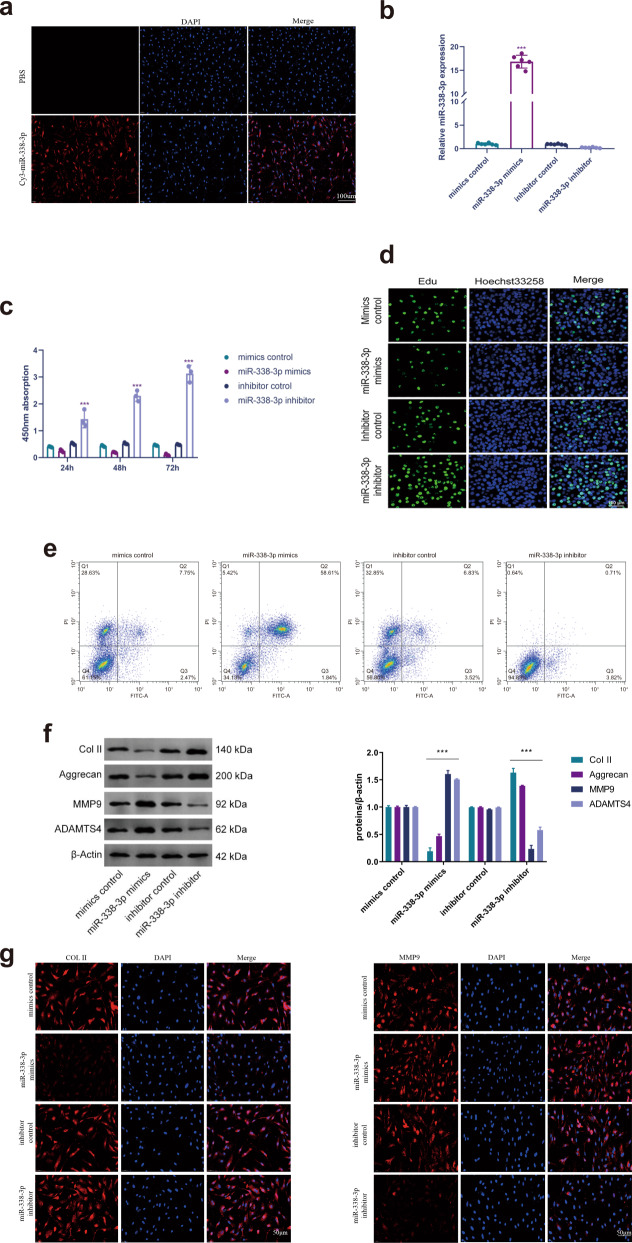
Functional analysis of miR-338-3p. **a** Transfection of Cy3 oligonucleotide-labeled miR-338-3p into cultured human NP cells. (scale bar = 100 μm). **b** qRT-PCR showing the transfection efficiency of miR-338-3p transfection in human NP cells. **c**, **d** CCK-8 and EdU assays showing the level of cellular proliferation of human NP cells transfected with miR-338-3p mimics or inhibitor. (scale bar = 100 μm). **e** Flow cytometry showing the apoptosis rate of human NP cells that were transfected with miR-338-3p mimics or inhibitor. **f** The expression levels of Col II, Aggrecan, MMP9, and ADAMTS4 detected using western blotting. **g** Immunofluorescence analysis of Col II and MMP9 expression levels. (scale bar = 50 μm). ***P < 0.01.

To determine the influence of miR-338-3p on the etiopathogenesis of IDD, we found that transfection with miR-338-3p mimics or inhibitor affected the cellular proliferation and apoptosis of human NP cells. The high transfection efficiency of miR-338-3p was confirmed by Cy3-labeled fluorescence and qRT-PCR (Fig. 2a, b). At three different time periods (24 h, 48 h, and 72 h), the miR-338-3p inhibitor significantly activated the cellular proliferation of NP cells, whereas the miR-338-3p mimics significantly repressed the cellular proliferation of NP cells (Fig. 2c). Thus, to determine the effects of miR-338-3p on the proliferation of NP cells, an EdU assay was performed (Fig. 2d). Correspondingly, the transfection of miR-338-3p mimics significantly promoted the apoptosis of NP cells (Fig. 2e). However, the silencing of miR-338-3p notably upregulated the expression of ECM-related proteins (Col II and Aggrecan), whereas overexpression of miR-338-3p significantly enhanced the expression of matrix-degrading enzymes (MMP9 and ADAMTS4) (Fig. 2f). These observations were further supported by immunofluorescence assay (Fig. 2g). Thus, our findings illustrated that the silencing of miR-338-3p can promote the proliferation of NP cells and enhance matrix synthesis.

### SIRT6 is a target gene for miR-338-3p

**Fig. 3:**
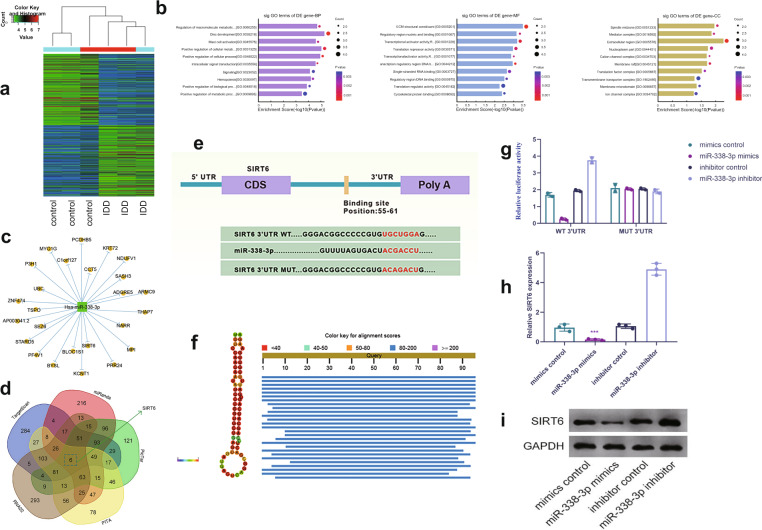
SIRT6 as a direct target gene of miR-338-3p. **a** Microarray analysis showing the differential gene expression between patients with IDD and normal controls. **b** Gene ontology analysis showing the highest enrichment scores of the upregulated GO terms. **c**, **d** Identification of SIRT6 as a potential regulatory target of miR-338-3p. **e** Sequential alignment showing the binding sites between miR-338-3p and SIRT6. **f** Computational alignment scores indicating high conservation of miR-338-3p. **g** Relative luciferase activity detection in human NP cells cotransfected with miR-338-3p mimic/inhibitor and SIRT6 3′UTR-wild/mutant-type reporter plasmids. **h**, **i** Analysis of the expression levels of SIRT6 mRNA and protein via qRT-PCR and western blotting following transfection with miR-338-3p mimics/inhibitor in human NP cells. ***P < 0.01.

A heat map was used to identify the dysregulated mRNAs in IDD (Fig. 3a). Gene ontology analysis predicted the functional characterization of downregulated genes, including those involved in disc development, ECM structural constituency, and extracellular region (Fig. 3b). Based on the miRNA–mRNA network and Venn analysis, SIRT6 was identified as a target gene for miR-338-3p (Fig. 3c–e). Furthermore, the computational alignment scores indicated a high conservation of miR-338-3p among various animal species (Fig. 3f). The luciferase reporter assay confirmed that miR-338-3p mimics can inhibit the luciferase activity of the wild-type SIRT6-3′ UTR. In contrast, the miR-338-3p mimics failed to inhibit the luciferase activity of the mutant SIRT6-3′ UTR (Fig. 3g). Our results thus indicated that miR-338-3p can directly bind to the SIRT6 3′ UTR, which suppressed the mRNA and protein expression levels of SIRT6 in human NP cells (Fig. 3h, i).

### miR-338-3p regulates IDD by modulating the SIRT6/MAPK/ERK signaling pathway

**Fig. 4:**
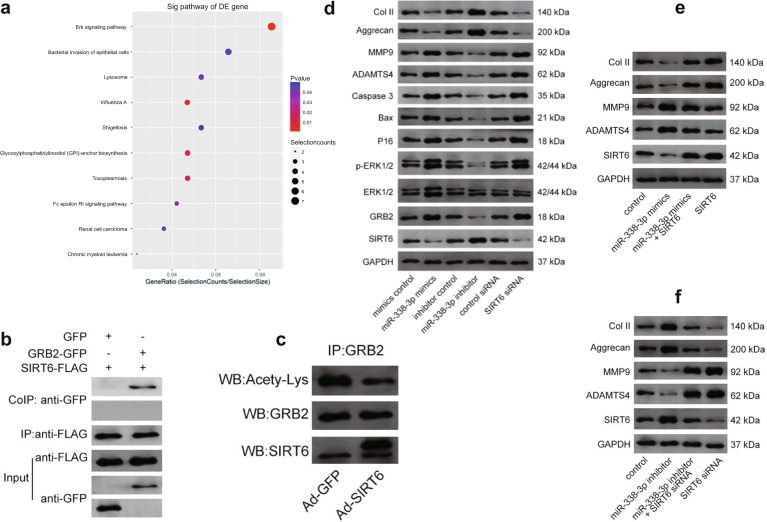
The modulation of miR-338-3p on the SIRT6/MAPK/ERK signaling pathway. **a** KEGG analysis showing the MAPK/ERK signaling pathway among the pathways most enriched with miRNAs upregulated in IDD samples. **b**, **c** Coimmunoprecipitation showing interactions between SIRT6 and GRB2. The deacetylation of GRB2 by SIRT6 results in its increased expression. **d** The expression levels of the proteins SIRT6, GRB2, ERK1/2, p-ERK1/2, P16, Bax, Caspase 3, Col II, Aggrecan, MMP9, and ADAMTS4 in human NP cells treated with miR-338-3p mimics/inhibitor and their negative controls or SIRT6 siRNA/control siRNA. **e** The transfection of miR-338-3p mimics resulted in decreased expression levels of Col II and Aggrecan, whereas restoration of SIRT6 expression reversed this effect. Similarly, SIRT6 overexpression inhibited MMP9 and ADAMTS4 expression, whereas miR-338-3p mimics reversed this decrease. **f** miR-338-3p inhibitor increased Col II and Aggrecan expression, whereas silencing SIRT6 blocked this effect. Similarly, SIRT6 knockdown increased MMP9 and ADAMTS4 expression, whereas the miR-338-3p inhibitor prevented this increase.

KEGG pathway analysis identified the MAPK/ERK signaling pathway as the most significantly enriched pathway (Fig. 4a). Importantly, coimmunoprecipitation showed that SIRT6 can directly deacetylate GRB2, which is a key component upstream of the MAPK/ERK signaling pathway (Fig. 4b, c). Thus, we hypothesized that SIRT6 can inhibit the transactivation capacity of GRB2, leading to the suppression of the MAPK/ERK pathway. The overexpression of miR-338-3p significantly increased GRB2, p-ERK1/2, cellular senescence-associated protein (P16), apoptosis-associated protein (Bax, Caspase 3), ADAMTS4, and MMP9 activities (Fig. 4d). We determined the possible direct relationship between miR-338-3p and SIRT6 using the levels of anabolic/catabolic markers such as Col II, Aggrecan, MMP9, and ADAMTS4 (Fig. 4e, f). These effects were maintained upon SIRT6 silencing according to the findings from the western blot, flow cytometry, and EdU assays (Fig. 4d, Supplementary Fig. 1 and 2). Furthermore, the functional role of miR-338-3p in the MAPK/ERK signaling pathway was also investigated. miR-338-3p mimics significantly upregulated the expression of MAPK/ERK signaling pathway-dependent proteins, including p-ERK1/2, ERK1/2, MEK2, MEK1, Raf, and Ras, whereas the miR-338-3p inhibitor significantly downregulated the expression of these proteins (Supplementary Fig. 3). Our results revealed that the inhibition of SIRT6 induced by miR-338-3p was able to trigger cellular senescence and apoptosis and promote IDD via the MAPK/ERK pathway.

### Intradiscal delivery of an miR-338-3p inhibitor attenuates IDD development

**Fig. 5:**
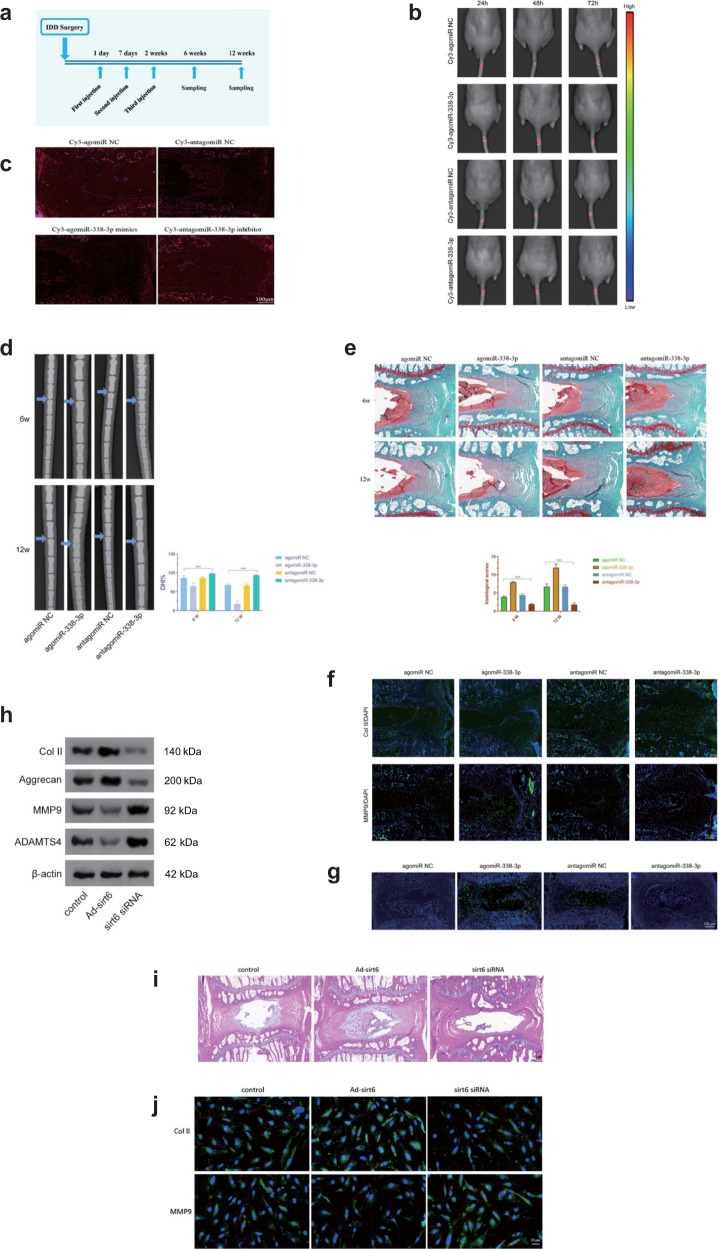
The therapeutic potential of miR-338-3p in IDD. **a** Schematic diagram of the experimental design: intradiscal delivery of miR-338-3p mimics, mimic control, miR-338-3p inhibitor, or control in mouse models on the 1^st^, 7^th^, and 14^th^ days post IDD surgery. **b**, **c** In vivo fluorescence imaging at 24 h, 48 h, and 72 h post intradiscal injection of Cy3 antagomir-338-3p, Cy3 agomir-338-3p, or their negative controls (scale bar = 100 μm). **d**, **e** The evaluation of the severity of IDD via the radiographical and histological findings at the 6^th^ and 12^th^ weeks post IDD surgery. The intervertebral discs of mice treated with antagomir-338-3p showed a significantly improved DHI% and histological score (scale bar = 50 μm). **f** Immunostaining showing the expression of Col II and MMP9 in mouse models treated with ago- and antagomir-338-3p 12 weeks post IDD surgery. (Scale bar = 50 μm). **g** TUNEL staining showing the degree of apoptotic activity in intervertebral discs of the IDD model treated with ago- and antagomir-338-3p at 12 weeks (scale bar = 100 μm). **h** The expression levels of the proteins Col II, Aggrecan, MMP9, and ADAMTS4 in mouse models injected with Ad-sirt6 or sirt6 siRNA or Ad-GFP. **i** Alcian blue staining of the intervertebral disc obtained from IDD mice eight weeks after adenovirus injection. The intervertebral discs of mouse models injected with sirt6 siRNA show loss of disc height and destruction of NP. Injection of Ad-sirt6 reversed this trend (scale bar = 500 μm). **j** Immunofluorescence assay showing the expression of Col II and MMP9 in mouse models injected with Ad-sirt6, sirt6 siRNA, or Ad-GFP eight weeks post adenovirus injection. (scale bar = 20 μm). ***p < 0.01.

Following the elucidation of the effect of miR-338-3p on the etiopathogenesis of IDD, in a first step toward translation, we sought to investigate its possible therapeutic effect on an IDD mouse model after intradiscal injection of an miR-338-3p inhibitor. Therapeutic testing was performed via local injection of injury-induced IDD mice with miR-338-3p (antagomir-338-3p or agomir-338-3p) on the 1^st^, 7^th^, and 14^th^ postoperative days (Fig. 5a). Live imaging of mice was used to monitor the in vivo disc-targeted ability of antagomir-338-3p or agomir-338-3p, and Cy3-labeled analysis was used to confirm the efficiency of the in vivo delivery of antagomir-338-3p or agomir-338-3p into the intervertebral disc (Fig. 5b, c). According to radiographic evaluation^27^, IDD mice treated with antagomir-338-3p exhibited a higher percentage of disc height index (DHI) (Fig. 5d). The intradiscal delivery of antagomir-338-3p significantly attenuated both cellular and morphological degeneration of AF and NP, according to the modified histological grading system^27–29^ (Fig. 5e). These radiographic and histologic findings suggested that miR-338-3p silencing can exert a therapeutic influence on the development of IDD. In contrast, the treatment of IDD mice with agomir-338-3p markedly aggravated disc degeneration. Immunofluorescence demonstrated that administration of antagomiR-338-3p downregulated the expression of a catabolic marker (MMP9) and upregulated the expression of an anabolic marker (Col II) in NPs (Fig. 5f). These findings indicated that antagomiR-338-3p regulated the imbalance between degradation and synthesis of NPs. In line with the aforementioned findings, TUNEL staining confirmed the significantly decreased level of cellular apoptosis in the NP cells of IDD mice injected with antagomiR-338-3p (Fig. 5g). To validate the influence of SIRT6 on IDD development in animal experiments, IDD mice were injected with Ad-sirt6, sirt6 siRNA, and Ad-GFP. We used Ad-sirt6 to introduce and overexpress sirt6 in intervertebral discs. The western blotting results showed that the overexpression of sirt6 significantly increased Aggrecan and Col II expression but significantly decreased MMP9 and ADAMTS4 expression (Fig. 5h). Histological findings suggested that sirt6 silencing markedly aggravated the progression of IDD, while sirt6 expression reversed this trend (Fig. 5i). The immunofluorescence assays also supported this finding (Fig. 5j). In animal experiments, overexpression of SIRT6 (Ad-sirt6) had effects on anabolic and catabolic markers similar to the effects induced by the miR-338-3p inhibitor, indicating that antagomir-338-3p attenuated IDD progression by targeting SIRT6. Taken together, our findings indicated that the miR-338-3p inhibitor (antagomir-338-3p) can be used as a potential therapeutic agent that could alleviate and reverse IDD. (Supplementary Fig. 4).

## Discussion

To the best of our knowledge, our study (both in vitro and in vivo) is the first to report on the possible functional role that can be played by miR-338-3p in the search for a viable therapeutic target for IDD treatment. In accordance with miRNA expression profiling, we found that the expression levels of miR-338-3p were remarkably increased in degenerated human discs, which was further confirmed via qRT-PCR in an independent cohort. Notably, we found a noteworthy positive correlation between miR-338-3p expression and the severity of IDD. Thus, our results indicated that miR-338-3p can serve as a possible novel biomarker for the prognosis of IDD. Based on several computational tools, SIRT6 was determined to be the direct target of miR-338-3p. Furthermore, our results revealed that the expression levels of SIRT6 were downregulated in degenerated NPs and that miR-338-3p can directly interact with SIRT6. Our findings also showed an inverse relation between miR-338-3p and SIRT6 and demonstrated that both miR-338-3p and SIRT6 may be important mediators in the pathogenesis of IDD.

SIRT6 is a member of the sirtuin family of deacetylases that are involved in diverse pathologies, including neurodegeneration, metabolic homeostasis, and aging^30–32^. The link between SIRT6 and MAPK signaling has been well reported in multiple systems. For instance, SIRT6 has been reported to protect against hepatic ischemia/reperfusion injury by regulating the transcriptional status of MAPK/ERK signaling-related genes^33^. Recent studies have provided evidence that increased activation of the MAPK signaling pathway promotes disc degeneration^34–36^. However, the possible functional role of SIRT6 deacetylase activity in the MAPK signaling pathway is yet to be fully understood. We hypothesize that SIRT6 may be involved in influencing the development of IDD through the MAPK signaling pathway and that its deacetylase activity is essential in engaging this process. Our study is possibly the first to identify that SIRT6 can modulate MAPK/ERK signaling via transcriptional control of GRB2, which is an upstream activator of this signaling pathway. From the results of our coimmunoprecipitation study, we observed that the enhanced expression of GRB2 was likely a direct consequence of the deacetylating activity of SIRT6. Furthermore, our findings revealed that the upregulation of GRB2 expression in NP cells led to a significant increase in the expression of target proteins in MAPK/ERK signaling (*p*-ERK1/2), as well as those involved in cellular senescence or apoptosis (P16, Bax, and Caspase 3), ultimately resulting in the upregulation of the expression levels of MMP9 and ADAMTS4 and downregulation of the expression of Col II and Aggrecan. The modulation of the MAPK signaling pathway induced by SIRT6 siRNA or the miR-338-3p mimics is possible via the regulation of GRB2 expression. Nevertheless, our findings do not exclude the possibility that other genes are involved in the control of MAPK/ERK signaling. Deeper exploration into the understanding of the mechanism by which miR-338-3p regulates the expression of SIRT6 and GRB2 is needed in future studies.

Our study provides a novel perspective for the clinical translation of miR-338-3p as a diagnostic and treatable option for IDD. The increased expression of miR-338-3p observed in IDD patients and animal models signified that miR-338-3p can be used as a stable biomarker for this common musculoskeletal disease. Our in vitro study revealed that in the absence of miR-338-3p, the expression of SIRT6 was upregulated and the MAPK/ERK signaling complex was suppressed, resulting in a reduced apoptosis rate of NP cells. Our results therefore indicated the viability of using miR-338-3p as a possible direct therapeutic target for reversing the development of IDD. In our preclinical study, we found that antagomir-338-3p (an miR-338-3p inhibitor) protected intervertebral discs against the typical pathological pattern of IDD by inhibiting the apoptosis of NP cells, enhancing the expression of extracellular matrix proteins (Col II and Aggrecan) and inhibiting the expression of extracellular matrix-degrading enzymes (MMP9 and ADAMTS4). These findings suggest that the possible underlying therapeutic mechanism of miR-338-3p inhibition is reversing the imbalance between catabolic and anabolic factors involved in IDD development. Collectively, our study introduces a new option for miR-338-3p-based therapeutics in IDD prevention.

In summary, our study revealed that miR-338-3p was significantly increased in human degenerative NPs and involved in the direct targeting of SIRT6, leading to the activation of the MAPK/ERK signaling pathway in NP cells. Our results showed that antagomir-338-3p, an miR-338-3p inhibitor, can reverse IDD development. Thus, miR-338-3p inhibitors can be used as possible novel agents for the therapeutic intervention of IDD.

## Supplementary information

Supplementary Information
