# Supplementary material for: MicroRNA-338-3p as a novel therapeutic target for intervertebral disc degeneration
Source: Exp Mol Med. 2021 Sep 16;53(9):1356–65. doi: 10.1038/s12276-021-00662-3 (PMC8492655; doi:10.1038/s12276-021-00662-3)
Supplement: Supplementary file 1 — Supplementary Information [file 12276_2021_662_MOESM1_ESM.docx]

**MicroRNA-338-3p as a novel therapeutic target for intervertebral disc degeneration**

**Hua Jiang *et al.***


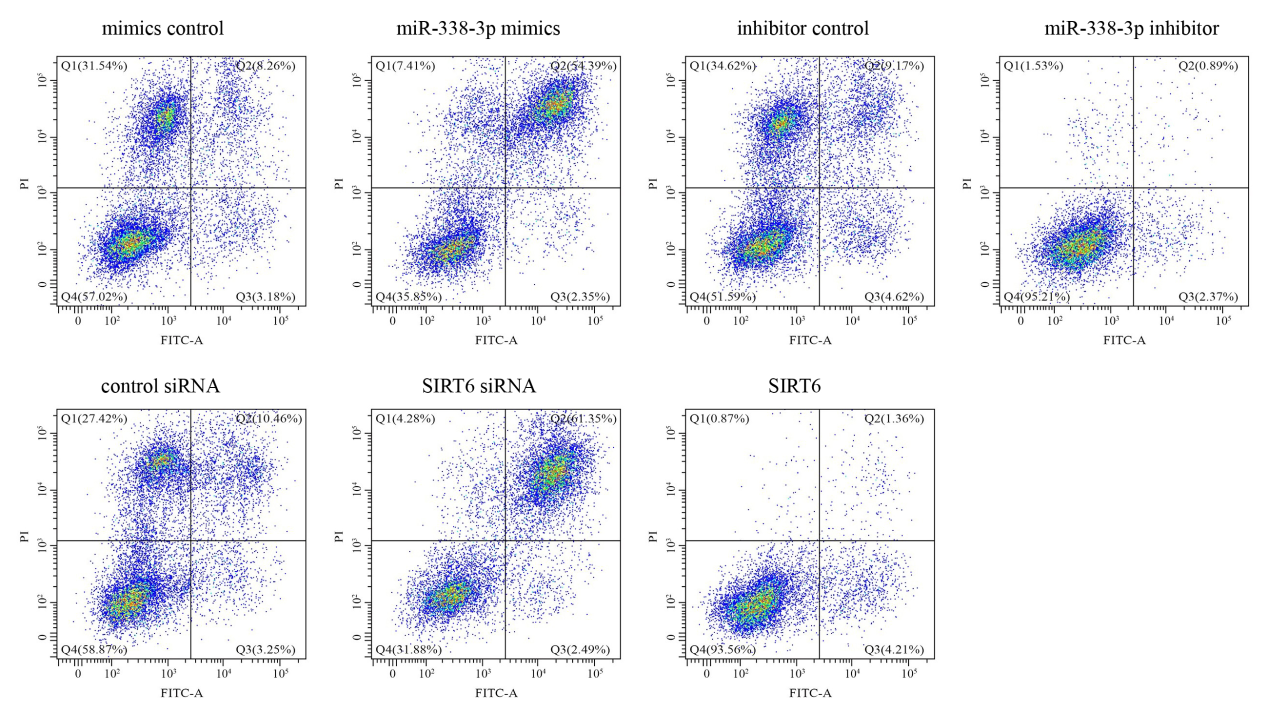


**Supplementary Figure 1**

Flow cytometry showing apoptosis of human NP cells that were transfected into miR-338-3p mimics/inhibitor and their negative controls, SIRT6, SIRT6 siRNA and control siRNA.


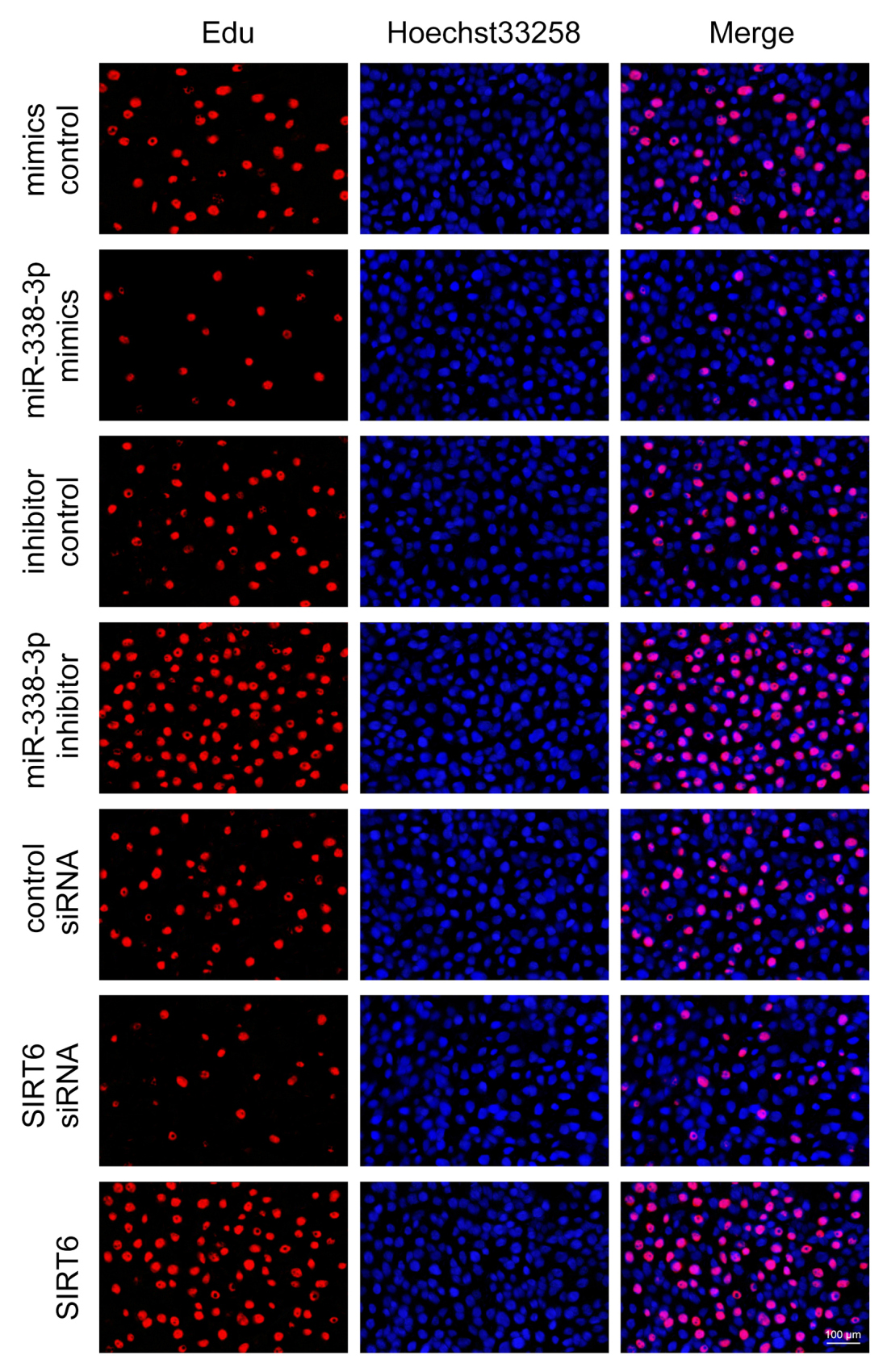


**Supplementary Figure 2**

EdU assays showing the level of cellular proliferation in human NP cells transfected with miR-338-3p mimics/inhibitor and their negative controls, SIRT6, SIRT6 siRNA and control siRNA. (Scale bar = 100 μm)


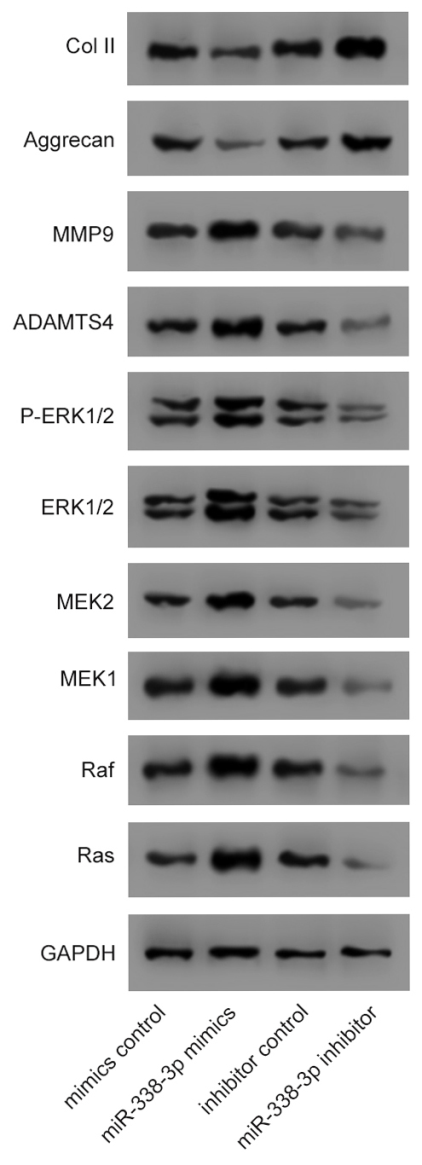


**Supplementary Figure 3**

The expression levels of the proteins Col II, Aggrecan, MMP9, ADAMTS4, p-ERK1/2, ERK1/2, MEK2, MEK1, Raf, and Ras in human NP cells treated with miR-338-3p mimics/inhibitor and their negative controls.


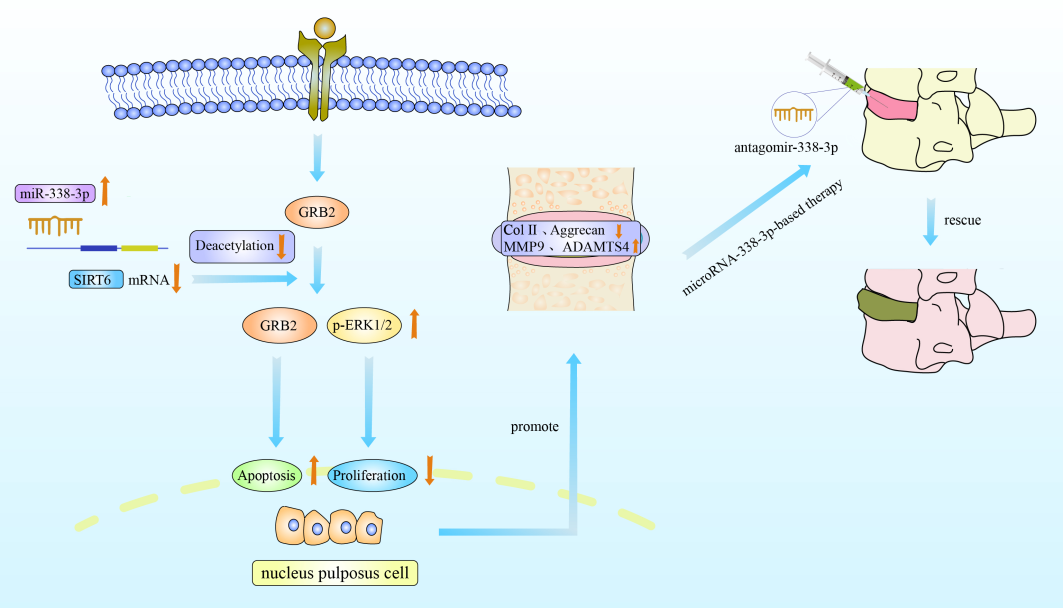


**Supplementary Figure 4 Preclinical development of a microRNA-based therapy for IDD**

Our studies found that miR-338-3p induced IDD by promoting NP cell catabolism and apoptosis via SIRT6/MAPK/ERK signaling pathway. Furthermore, we tested whether intra-discal injection of miR-338-3p inhibitor exhibited NP-protective effects in IDD mice models.

| **Supplementary Table 1 Primer sequences used for qRT-PCR analysis** | | |
| --- | --- | --- |
| **Gene** | **Primer orientation** | **Primer sequence (5’-3’)** |
| miR-338-3p | Forward | TGCGGTCCAGCATCAGTGAT |
|  | Reverse | CCAGTGCAGGGTCCGAGGT |
| SIRT6 | Forward | CCCACGGAGTCTGGACCAT |
|  | Reverse | CTCTGCCAGTTTGTCCCTG |
| Col II | Forward | CCTGGCAAAGATGGTGAGACAG |
|  | Reverse | CCTGGTTTTCCACCTTCACCTG |
| Aggrecan | Forward | TGAGGAGGGCTGGAACAAGTACC |
|  | Reverse | GGAGGTGCTAATTGCAGGGAACA |
| MMP9 | Forward | GCCACTACTGTGCCTTTGAGTC |
|  | Reverse | CCCTCAGAGAATCGCCAGTACT |
| ADAMTS4 | Forward | TCACTGACTTCCTGGACAATGGC |
|  | Reverse | GGTCAGCATCATAGTCCTTGCC |
| GAPDH | Forward | GTCTCCTCTGACTTCAACAGCG |
|  | Reverse | ACCACCCTGTTGCTGTAGCCAA |

| **Supplementary Table 2 Differentially expressed miRNAs in NP tissues from IDD and controls in both one- and two-stage validation** | | | | |
| --- | --- | --- | --- | --- |
| **miRNAs** | **One-stage** | | **Two-stage** | |
|  | **Fold change** | **P-value** | **Fold change** | **P-value** |
| **Up-regulated** |  |  |  |  |
| Hsa-miR-224-5p | 5.6 | 0.08 | - | - |
| Hsa-miR-510-5p | 2.7 | 0.12 | - | - |
| Hsa-miR-147a | 5.3 | 0.09 | - | - |
| Hsa-miR-208-3p | 4.8 | 0.16 | - | - |
| **Hsa-miR-338-3p** | **7.8** | **0.001**** | **7.5** | **0.002**** |
| Hsa-miR-371a-3p | 3.6 | 0.06 | - | - |
| Hsa-miR-577 | 6.2 | 0.18 | - | - |
| Hsa-miR-451 | 5.1 | 0.25 | - | - |
| **Hsa-miR-198** | **7.2** | **0.006**** | 5.6 | 0.15 |
| Hsa-miR-1224-5p | 4.9 | 0.37 | - | - |
| Hsa-miR-345-5p | 4.6 | 0.19 | - | - |
| Hsa-miR-676-3p | 3.7 | 0.22 | - | - |
| Hsa-miR-802 | 5.9 | 0.05 | - | - |
| Hsa-miR-489-3p | 5.3 | 0.06 | - | - |
|  |  |  |  |  |
| **Down-regulated** |  |  |  |  |
| Hsa-miR-660-5p | 0.15 | 0.12 | - | - |
| **Hsa-miR-874-3p** | **0.02** | **0.005**** | 0.04 | 0.09 |
| Hsa-miR-202-5p | 0.12 | 0.18 | - | - |
| Hsa-miR-193a-5p | 0.31 | 0.35 | - | - |
| Hsa-miR-599 | 0.28 | 0.42 | - | - |
| Hsa-miR-642a-5p | 0.09 | 0.06 | - | - |
| Hsa-miR-500a-3p | 0.10 | 0.23 | - | - |
| Hsa-miR-5094 | 0.23 | 0.33 | - | - |
| Hsa-miR-98 | 0.06 | 0.13 | - | - |
| NP: nucleus pulposus; IDD: intervertebral disc degeneration; Hsa: human; ** P < 0.01 by  Mann-Whitney U test. | | | | |
